# Relapsing polychondritis with isolated tracheal involvement and airway‐only symptoms

**DOI:** 10.1002/rcr2.651

**Published:** 2020-08-31

**Authors:** Sheng‐Yuan Wang, Chia‐Tse Weng, Lili Cheng, Tang‐Hsiu Huang

**Affiliations:** ^1^ Division of Chest Medicine, Department of Internal Medicine National Cheng Kung University Hospital, College of Medicine, National Cheng Kung University Tainan Taiwan; ^2^ Division of Rheumatology and Immunology, Department of Internal Medicine National Cheng Kung University Hospital, College of Medicine, National Cheng Kung University Tainan Taiwan; ^3^ Department of Diagnostic Radiology National Cheng Kung University Hospital, College of Medicine, National Cheng Kung University Tainan Taiwan

**Keywords:** Bronchoscopy, central airway obstruction, tracheal stenosis

## Abstract

Relapsing polychondritis (RP) is a rare autoimmune disorder, characterized by the inflammation of cartilaginous structures and proteoglycan‐rich tissues. Due to its rarity and the notoriously variable presentations, the diagnosis of RP could be challenging. We report an unusual case of RP with isolated tracheal involvement and very non‐specific symptoms of exertional dyspnoea and dry cough. The initial chest radiograph showed long‐segment narrowing of the trachea, and the computed tomography of the chest revealed thickened cartilaginous walls of the trachea, while the posterior membranous portion was spared. The tracheal narrowing was readily observed under bronchoscopy. The patient was treated with oral prednisolone. Although the subsequent course was transiently complicated by an episode of severe *Pneumocystis jirovecii* pneumonia with acute respiratory distress syndrome, the patient overall responded well to systemic corticosteroid therapy. No new symptoms developed during a two‐year follow‐up.

## Graphical abstract

We describe an atypical case of relapsing polychondritis that involved solely the tracheal cartilages and presented clinically as non‐specific airway symptoms. Timely diagnosis and treatment through a multidisciplinary approach is crucial to prevent subsequent serious complications.

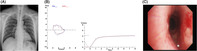

## Introduction

Relapsing polychondritis (RP) is a rare multi‐system autoimmune disease. Chondritis (especially auricular and nasal) and polyarthritis are the most common presentations, but the eyes, heart valves, skin, and blood vessels can also be affected [1]. Because of such clinical variations, RP has been associated with a diagnostic delay. Laryngo‐tracheo‐bronchial involvement eventually occurs in 50% of RP patients, leading to significant morbidity and mortality, but it is uncommon as the initial presentation [2, 3]. In this report, we describe an unusual case of RP that had tracheal chondritis as the initial and main manifestation.

## Case Report

**Figure 1:**
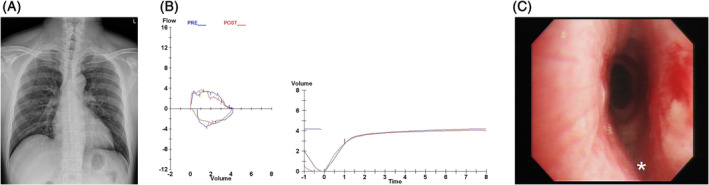
(A) The initial chest radiograph showed evident narrowing of both the extra‐ and intra‐thoracic portions of the trachea. (B) The flow–volume revealed a flattened curve in both the inspiratory and expiratory phases, suggesting a fixed central airway obstruction. (C) Bronchoscopic examination showed severe narrowing and loss of integrity of the cartilaginous rings throughout the mid‐to‐lower portion of the trachea (the asterisk marks the posterior membranous surface of the trachea).

**Figure 2:**
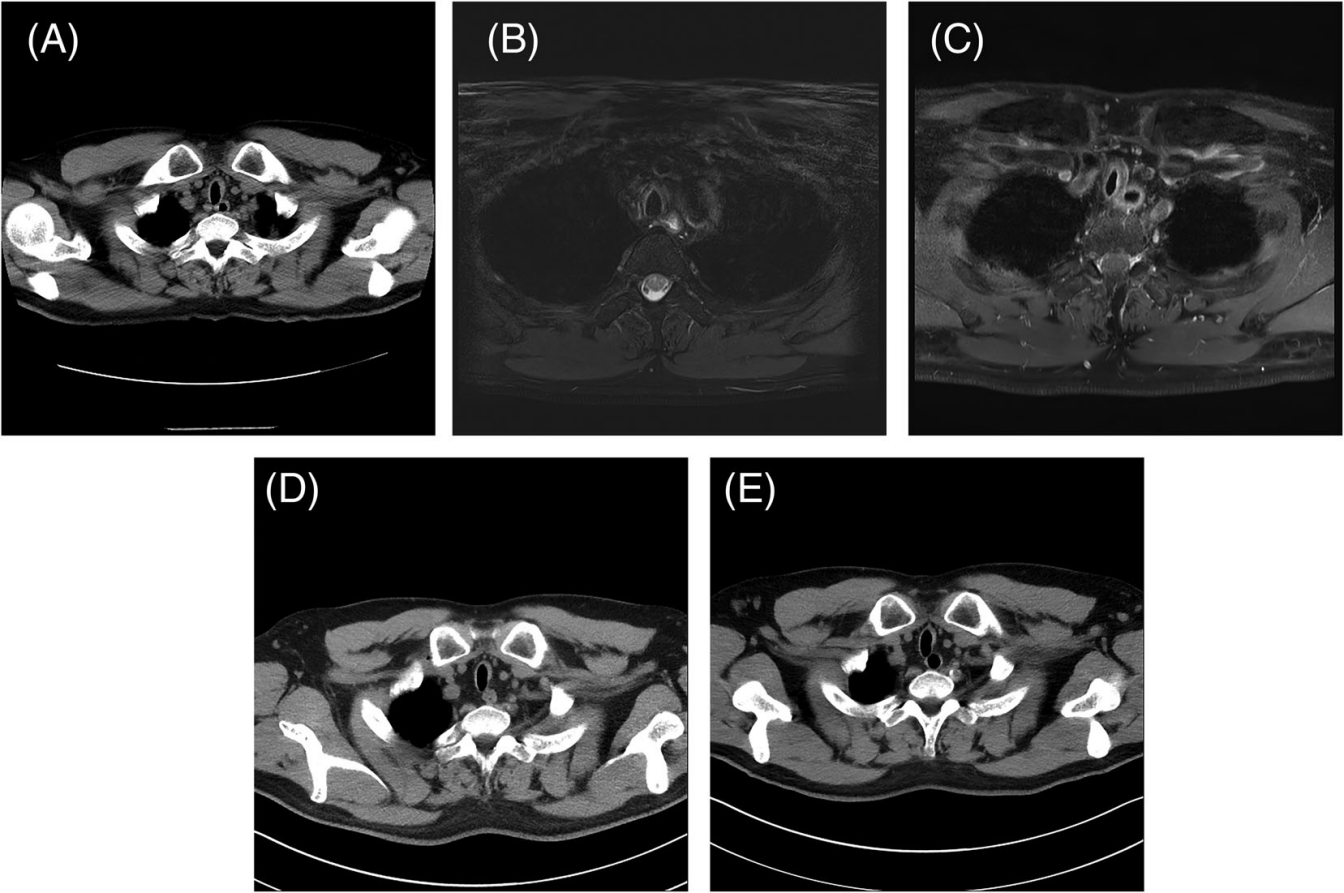
(A) Computed tomography (CT) of the chest revealed diffuse thickening of the cartilaginous portion of the trachea, with the posterior membrane spared. (B) Magnetic resonance imaging (MRI) of the chest showed that the periphery of the tracheal cartilaginous C‐ring exhibited hyperintensity on the T2‐weighted image and (C) enhancement on the post‐gadolinium T1‐weighted image. (D) Five months into treatment, follow‐up CT showed decreased thickness of the cartilaginous wall of the trachea, which remained stable at 10 months as shown in (E).

The patient was a 47‐year‐old man, who initially presented to a cardiologist with exertional dyspnoea and dry cough for several months. There was no fever, no neck or chest pain, no haemoptysis, no joint pain or skin eruption, and no visual, nasal, or auditory complaints. He did not have abdominal discomfort, chronic diarrhoea, or haematochezia. His childhood growth and past medical history were unremarkable. He was an ex‐smoker and worked as an operator of forklift at a warehouse. Initial workups including the electrocardiogram, the echocardiography, a treadmill stress test, and a thallium‐201 myocardial scan were normal. He was treated with aspirin and beta‐blocker, but the symptoms persisted. Upon the combined examination by a pulmonologist and a rheumatologist six months later, neither stridor nor wheeze was auscultated. Ophthalmologic, auricular, and nasopharyngeal examinations were unremarkable; his hearing test was normal. However, the initial chest radiograph showed evident narrowing of both the extra‐ and intra‐thoracic trachea (Fig. 1A). The pulmonary spirometry also detected an obstructive ventilatory deficit (Table 1), wherein a “flattening curve” was observed in both the inspiratory and expiratory phases (Fig. 1B), suggesting a fixed central airway obstruction. Computed tomography (CT) of the chest revealed diffuse thickening of the cartilaginous portion of his trachea that spared the posterior membranous portion (Fig. 2A); there was not enlarged lymph node in the cervical, clavicular, or mediastinal regions. Although an elevated erythrocyte sedimentation rate (ESR: 50, normal range: 0–15, mm/h) was found, other pertinent blood workups (including the haemogram, renal and hepatic parameters, the albumin‐to‐globulin ratio, and levels of C‐reactive protein, creatinine kinase, rheumatoid factor, immunoglobulins, and selected autoantibodies; Table 2) reported normal values. A gallium‐67 inflammation scan also revealed no definite inflammatory focus. Under fibreoptic bronchoscopy, significant narrowing of the mid‐to‐lower portion of the trachea was readily observed, but without excessive invagination or outpouching of the posterior wall. The narrowed lumen was neither crescent‐shaped nor sabre‐sheath‐like in the transverse view, and the mucosal surface was smooth without nodularity (Fig. 1C, Video S1, Supporting Information). A subsequent magnetic resonance imaging (MRI) showed hyperintensity and post‐gadolinium enhancement typically involving the periphery of the cartilaginous portions of the trachea (Fig. 2B, C). The diagnosis of RP was established following multidisciplinary evaluation and discussion, and treatment with high‐dose oral prednisolone (1 mg/kg/day) plus methotrexate (15 mg/week) was initiated. Just three weeks into treatment, the patient had an episode of severe *Pneumocystis jirovecii* pneumonia (PJP), which was diagnosed based on the typical radiographic findings of bilateral ground‐glass opacity and the detection of *P. jirovecii* in the deep tracheal aspirate (using an automated quantitative real‐time polymerase chain reaction (PCR) platform from Becton Dickinson in the central laboratory). The disease led to acute respiratory distress syndrome (ARDS) within three days. Methotrexate was discontinued immediately, but systemic steroid was maintained (and adjusted) as part of the treatment for PJP. After 13 days of invasive mechanical ventilation, the patient was successfully extubated and recovered well afterwards. No other pathogen (including mycobacteria) was isolated from his lower airway specimens. Over the next five months, corticosteroid therapy was carefully tapered to 2.5–5 mg daily of oral prednisolone, with a prophylaxis against PJP. His exertional dyspnoea and cough gradually improved, and he was able to return to work. CT of the chest five months later revealed decreased thickness of the tracheal cartilages, which remained stable at 10 months (Fig. 2D, E). The follow‐up pulmonary function test showed a significant improvement in his obstructive deficit (Table 1), and serial ESR levels were within normal ranges (Table 2). Two years had passed since the first onset of his symptoms, and the patient still receives active surveillance and has not developed new symptoms.

**Table 1:** Serial measurements of pulmonary spirometry of the patient.

	FEV_1_/FVC, %	Pre‐BD FEV_1_, L (% prediction)	Post‐BD FEV_1_, L (% prediction)	FVC, L (% prediction)	Notes
October 2018	66	2.77 (80)	2.79 (80)	4.17 (107)	The initial spirometry before treatment
August 2019	81	3.11 (90)	3.06 (88)	3.86 (99)	Post‐treatment spirometries (corticosteroid treatment started in May 2019)
February 2020	83	3.18 (93)	NA	3.85 (99)	

BD, bronchodilator; FEV_1_, forced expiratory volume in 1 sec; FVC, forced vital capacity; NA, not available.

**Table 2:** Data on the baseline blood tests of the patient.

Haemogram		Reference range
Total WBC count	3.5	3.2–9.2 x 10^3^/μL
Total RBC count	3.48	3.72–4.93 x 10^6^/μL
Total platelet count	198	151–366 x 10^3^/μL
Hb	11.4	11.6–14.8 g/dL
Hct	33.8	33.8–43.4%
Blood biochemistry		
Creatinine	0.50	0.5–0.9 mg/dL
AST	33	10–35 U/L
ALT	23	10–35 U/L
Albumin	4.0	3.5–5.0 g/dL
Total protein	7.4	6.4–8.3 g/dL
CK	124	37–308 U/L
Inflammation‐associated markers		
LDH	185	135–225 U/L
ESR		0–15 mm/h
20 June 2018	50	
24 June 2019	10	
6 March 2020	6	
CRP	<7	0.0–8.0 mg/L
Ig		
IgG	1120	750–1690 mg/dL
IgA	415	82–463 mg/dL
IgM	64.5	46–304 mg/dL
IgE	10.3	0–100 U/mL
Auto‐antibodies		
ANA	1:40	≤1:40
RF	<20	<20 U/mL
Anti‐CCP Ab	0.6	<7 U/mL
Anti‐cardiolipin IgG	0.7	<10 U/mL
Anti‐cardiolipin IgM	2.0	<10 U/mL
Anti‐SmDP Ab	0.7	<7 U/mL
Anti‐RNP Ab	0.5	<5 U/mL
Anti‐SSA (Ro) Ab	0.3	<7 U/mL
Anti‐SSB (La) Ab	0.2	<7 U/mL
Ant‐MPO Ab	0.1	<3.5 U/mL
Anti‐PR3 Ab	0.1	<2 U/mL

Ab, antibody; ALT, alanine transaminase; ANA, anti‐nuclear antibody; AST, aspartate transaminase; CCP, cyclic citrullinated peptide; CK, creatinine kinase; CRP, C‐reactive protein; ESR, erythrocyte sedimentation rate; Hb, haemoglobin; Hct, haematocrit; Ig, immunoglobulin; LDH, lactic dehydrogenase; MPO, myeloperoxidase; PR3, proteinase 3; RBC, red blood cell; RF, rheumatoid factor; RNP, ribonucleoprotein; SmDP, Smith protein D; SSA, Sjogren's‐syndrome‐related antigen A; SSB, Sjogren's‐syndrome‐related antigen B; WBC, white blood cell.

## Discussion

RP is a rare autoimmune disease, with reported prevalence rates ranging from 4.5 to 9 cases per million persons [1, 4, 5, 6]. RP is difficult to diagnose due to the rarity, the episodic nature, and its diverse clinical presentation. Significant delays in diagnosis have been described [4, 7, 8, 9]. Currently, these are still no standardized guidelines for the diagnosis of RP. Several sets of diagnostic criteria, which are mainly based on the presence of symptoms suggesting chondritis or arthritis at multiple sites, have been proposed previously [9, 10, 11, 12, 13]. These sets of criteria, however, have limitations in terms of sensitivity and accuracy, as was demonstrated by recent retrospective case analyses [9, 10]. Histologic confirmation has been included into the criteria proposed by Demiani and Levine [12], but the histologic findings are not specific, and the site of inflammation is not always safe for biopsy. Moreover, although chondritis of the airway eventually develop in up to 50% of patients, only 10% of patients have symptoms or signs suggesting airway involvement at presentation [3, 7]. A recent large case series reported that only 4% and 3.5% of patients had cough and dyspnoea, respectively, as their initial symptoms [14]. Hazra et al. reported that respiratory symptoms were associated with the longest delay in diagnosis, with a median delay time of 10.4 years, while leaving 64% of patients pending diagnosis for more than two years [5]. The presence of respiratory symptoms in a patient known to have RP raises the clinical suspicion of airway involvement [15], but it would be challenging to establish the diagnosis of RP in someone presenting only with cough and dyspnoea. Considering the non‐specific and airway‐only manifestation of our patient, it was not helpful to diagnose his RP by applying those aforementioned sets of criteria. Besides, central airway biopsy was withheld for safety concerns, as the patient had already exhibited significant tracheal narrowing and respiratory deficit at the time of the bronchoscopy. Nevertheless, with a multidisciplinary approach integrating his past histories, symptoms, and serologico‐biochemical, bronchoscopic, radiographic, and spirometric findings, other diseases with similar clinico‐radiological presentations (such as tuberculous tracheitis, anti‐neutrophilic cytoplasmic autoantibodies (ANCA)‐associated vasculitis, sarcoidosis, amyloid deposition, tracheomalacia, excessive dynamic airway collapse, tracheal involvement in inflammatory bowel disease, rhinoscleroma, or tracheobronchopathia osteochondroplastica) were ruled out. Our patient received the correct diagnosis within 12 months after the onset of his symptoms, which might be considered a relatively short delay.

There is likely heterogeneity among patients with RP [1, 3, 14, 16]. Using cluster analysis, Dion et al. discovered that patients with RP could be categorized into three clinically relevant phenotypes, namely haematological, respiratory, and mild phenotypes [14]. The haematological phenotype and the mild phenotype represent the two extremes of the spectrum, with the former being associated with older ages of onset and high risks of concurrent myelodysplastic syndrome and mortality, and the latter, the best prognosis and the highest likelihood of long‐term remission. Patients with the respiratory phenotype appeared to have intermediate prognosis. They were reported to be younger and had predominantly airway involvement but less involvement of the heart and ears. Interestingly, they were also most likely to have received immunosuppressive agents and had high rates of infectious complications (35%) and intensive care admission (27%) [14]. Considering the overall clinical manifestations and disease course, our patient can be reasonably classified as having the respiratory phenotype.

Long‐term airway complications of RP, such as tracheomalacia or tracheobrochial stenosis, have been well documented. Around 35–40% of patients with known airway involvement eventually required some form of interventions, such as tracheostomy, balloon dilatation, stenting, or a combination [2, 3, 10]. Moreover, for patients with long‐standing RP, life‐threatening airway collapse could be the first manifestation to unveil occult airway involvement [15]. The treatment of RP is largely based on experiences from previous case reports and small series, and there are still no evidence‐based guidelines [1, 3, 17]. Corticosteroids and non‐steroidal anti‐inflammatory drugs (NSAIDs) were administered most frequently, particularly for those milder cases, while conventional disease‐modifying antirheumatic drugs (DMARDs) and newer biologics are usually used as steroid‐sparing agents or in severe scenarios [1, 17, 18]. Despite the infectious complication, our patient has overall responded well to the treatment with systemic corticosteroid, which has been gradually tapered to his current low‐maintenance dose (2.5–5 mg of prednisolone daily).

In conclusion, through this case report, we would like to emphasize that, although uncommon, RP could have an airway‐only manifestation, thereby leading to great diagnostic difficulties. The treatment should be tailored according to individual patient's organ involvement and complications, although no guidelines exist to date. RP patients with airway involvement have higher infection risks and high rates of long‐term airway complications, so it is pivotal to recognize and manage these airway morbidities in a timely fashion to prevent fatal consequences.

### Disclosure Statement

Appropriate written informed consent was obtained for publication of this case report and accompanying images.

## Supporting information


**Video S1.** Bronchoscopic examination of the patient before the initiation of corticosteroid treatment. This video, recorded during the bronchoscopic examination of the patient before the initiation of corticosteroid treatment, began at the lower trachea close to the main carina, and then moved slowly and proximally towards the vocal cords. The video displayed the persistent narrowing of the mid‐to‐lower trachea of the patient.
